# Articular surface integrity assessed by ultrasound is associated with biological characteristics of articular cartilage in early-stage degeneration

**DOI:** 10.1038/s41598-022-16248-6

**Published:** 2022-07-13

**Authors:** Wen Shi, Takashi Kanamoto, Masaharu Aihara, Shiro Oka, Sanae Kuroda, Tsuyoshi Nakai, Takeo Mazuka, Keisuke Takenaka, Yuji Sato, Masahiro Tsukamoto, Kosuke Ebina, Ken Nakata

**Affiliations:** 1 Department of Medicine for Sports and Performing Arts, Osaka University Graduate School of Medicine, 2-2 Yamadaoka, Suita, Osaka 565-0871 Japan; 2 Department of Orthopedic Surgery, Aihara Hospital, 3-4-30 Makiochi, Mino, Osaka 562-0004 Japan; 3 Department of Orthopedic Surgery, Itami City Hospital, 1-100 Koyaike, Itami, Hyogo 664-8540 Japan; 4 Department of Orthopedic Surgery, Hannan Chuo Hospital, 3-3-28 Minami-shinmachi Matsubara, Osaka, 580-0023 Japan; 5 Joining and Welding Research Institute, Osaka University, 11-1 Mihogaoka, Ibaraki, Osaka 567-0047 Japan; 6 Department of Musculoskeletal Regenerative Medicine, Osaka University Graduate School of Medicine, 2-2 Yamadaoka, Suita, Osaka 565-0871 Japan

**Keywords:** Diagnosis, Cartilage

## Abstract

Early diagnosis of articular cartilage damage and repeated evaluation of treatment efficacy are essential for osteoarthritis treatment. In this study, we established a simple ultrasound grading system for early degenerative articular cartilage and investigated its relationship with cartilage biological characteristics. The ultrasound grading system were based on surface integrity (S1a: continuous high-echo lines, S1b: discontinuous or weak high-echo lines, S2: surface irregular) and cartilage echogenicity (E1: with > 50%, E2: < 50% hypoechoic area of total cartilage layer) and verified by surface roughness (Ra; μm) and histological staining. Ra was lower in S1 than in S2, and the percentage of hypoechoic and safranin O-stained areas was positively correlated. Then we examined its relationship with histopathological evaluation (OARSI grade), gene expression, and protein production in responded to pro-inflammatory cytokine (IL-1ß) stimulation. OARSI grades were different among S grades. The superficial layer of S1 had higher expression of Collagen10, aggrecan, Sox9, and lower expression of Collagen1 and BMP2 than that of S2. S1 responded more pronouncedly to IL-1ß in IL-6, IL-8, and CCL2 production than S2. There was no difference among the E-grades. Taken together, our findings indicate that ultrasound assessment using surface integrity can reflect the biological characteristics of early degenerative articular cartilage.

## Introduction

Articular cartilage disorders caused by osteoarthritis (OA) and traumatic injuries are serious social and medical problems due to the number of patients affected and the impact these disorders on activities of daily living^1,2^. Studies on the pathogenesis of articular cartilage disorders have identified the molecules involved, such as Interleukin 1 beta (IL-1ß), tumor necrosis factor alpha (TNF-α), Interleukin 6 (IL-6), Interleukin-8 (IL-8), and Matrix metalloproteinases (MMPs), and many studies on therapeutic interventions are underway and expected to be successful, which may revolutionize treatment soon^3,4^. Currently, the treatment modalities with strong evidence of reducing the symptoms and progression of early OA are non-pharmacological therapies, such as weight control and appropriate exercise^1,5^. To properly initiate these treatments, a reliable early diagnosis of articular cartilage damage is essential. The existence of a tool that allows repeated evaluation of treatment effects, when necessary, would be of great benefit in terms of the selection of treatment modalities and patient compliance.

Generally, the diagnosis and evaluation of articular cartilage disorder is based on subjective symptoms, such as pain, physical findings (swelling or limited range of motion), and imaging findings (X-rays or MRI)^6–8^. Although subjective and physiological evaluations are critically important for OA diagnosis, these evaluations could be influenced by multiple factors, such as the patient’s background and doctor’s experience^9,10^. Therefore, more objective and quantitative evaluations are required. Currently, most biochemical evaluations of blood and synovial fluid for articular cartilage disorders are still in the research stage and are not commonly used in clinical practice, whereas imaging is frequently used for objective evaluation of articular cartilage disorders^7^. For the imaging evaluation of articular cartilage, there has been a great deal of clinical and basic research on magnetic resonance imaging (MRI)^11,12^. MRI has been proven to evaluate the cartilage within the entire joint with high resolution and can visualize abnormalities that are not present on radiography^7^. However, due to the high cost, long examination time, and limited availability of facilities, MRI is not suitable for OA screening and frequent longitudinal evaluation. Therefore, there is a high demand for a simple evaluation of articular cartilage damage, associated with OA progression, that can be performed multiple times.

Ultrasound (US), imaging modality, that is cost-effective, portable, real-time, non-invasive, and frequently used in clinical practice. It requires the examiner to have a relatively high level of operating skills, also the visualization of articular cartilage is limited^13,14^. In the case of knee joints, it cannot assess the whole area of the femoral condyle^15,16^. However, despite these problems, it is expected to have a great potential in the evaluation of OA cartilage depending on how it is used, because it can evaluate surface tissues with higher resolution than MRI^16,17^. In US images, healthy articular cartilage is detected as a homogeneous hypoechoic thin layer structure between the chondrosynovial and osteochondral margins at different synovial joints, including the knee, elbow, wrist, shoulder, tibiotalar, and metacarpophalangeal joints^14^. Previous studies have shown that US measurements of articular cartilage thickness mirror those assessed by MRI or contrast-enhanced micro-computed tomography (micro-CT)^16–18^. Furthermore, some under-researched US-assisted techniques such as quantitative US techniques and US elastography could show more mechanical properties of the cartilage, which may be applicable for arthroscopy or open surgery in the future^19,20^. Previous studies used surface topography and internal echogenicity as indicators of degenerative changes in articular cartilage, and these indicators have been found to reflect arthroscopic evaluation^15^. Currently, arthroscopy is often regarded as the gold standard for articular cartilage evaluation; however, it falls short of histological and molecular biological assessment in terms of investigating the biological properties of the tissue^21,22^. To the best of our knowledge, no study has examined the relationship between US findings of articular cartilage and biological characteristics such as gene expression, and response to external stimuli like inflammatory cytokines.

Thus, the identification of US findings that reflect the degenerative state of the articular cartilage and its response to external stimuli such as inflammatory cytokines will greatly contribute to the diagnosis and treatment strategies for OA. The purpose of this study was to establish a new US grading system for articular cartilage that is simple and reproducible, and to investigate the relationship between the grading and biological characteristics such as OA-related gene expression and response to IL-1ß stimulation.

## Results

### Histological analysis of surface integrity of articular cartilage and quantitative evaluation of surface roughness

**Figure 1:**
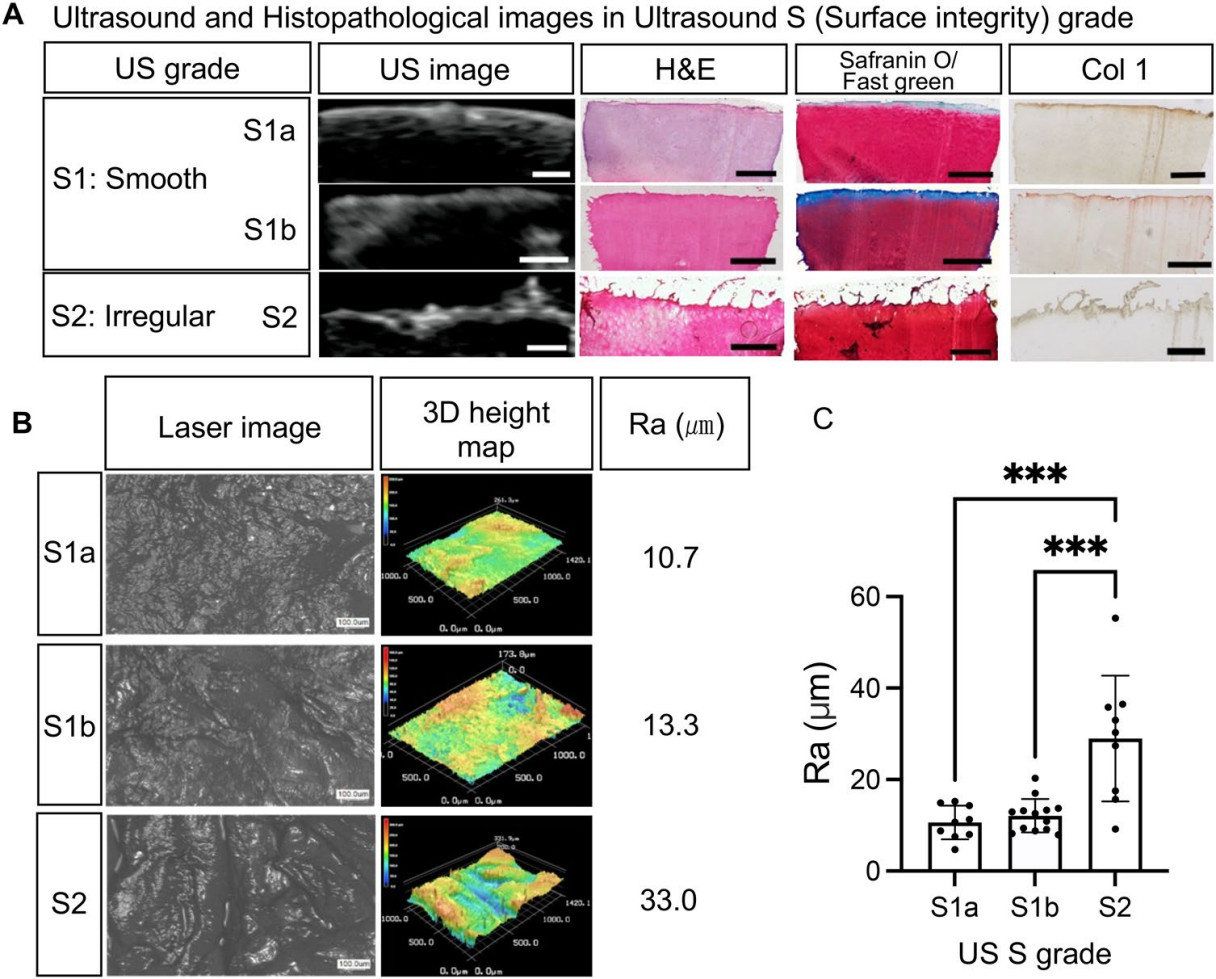
Ultrasound S (Surface integrity) grade and the surface roughness of articular cartilage. (**A**) Ultrasound images of typical cases. Comparing with S1, the cartilage surface integrity was lost in S2. Within S1, the superficial zones were basically intact, but in S1b there were more deep fibrillations than that of S1a. Scale bars: 1 mm. (**B**) Typical examples of surface roughness analysis. Scale bars: 100 μm. (**C**) Mean Ra; bars are show the mean ± SD. ***p < 0.001.

The cartilage surface was graded as S1 if it was smooth, S1a if there were continuous high-echo lines on the cartilage surface, and S1b if the high-echo lines were discontinuous or weak. If the cartilage surface was irregular, it was assessed as S2 (Fig. 1). Kappa statistics (K) between the two examiners were 0.849. The H&E staining, safranin O/fast green staining, and type 1 collagen (Col 1) immunostaining showed a difference in the cartilage surface condition between S grades. Compared to S1, the surface integrity was lost, and the continuity of Col 1 in the cartilage surface was lost in S2 (Fig. 1A). In addition, within S1, the superficial zones of S1a and S1b were basically intact in H&E staining and safranin O/fast green staining, but more surface discontinuity could be observed in S1b than S1a(Fig. 1A).

Surface roughness (Ra; μm) was measured for a total of 31 specimens obtained from four donors (Fig. 1B, C). The average Ra (mean ± SD) were 10.6 ± 3.7 (S1a), 12.1 ± 3.6 (S1b), and 29.0 ± 13.8 (S2). Compare with S2, Ra was lower in S1a (*p* = 0.0001) and S1b (*p* = 0.0001). There was no statistically significant difference between S1a and S1b (*p* = 0.902).

### Histological analysis of cartilage echogenicity of articular cartilage

**Figure 2:**
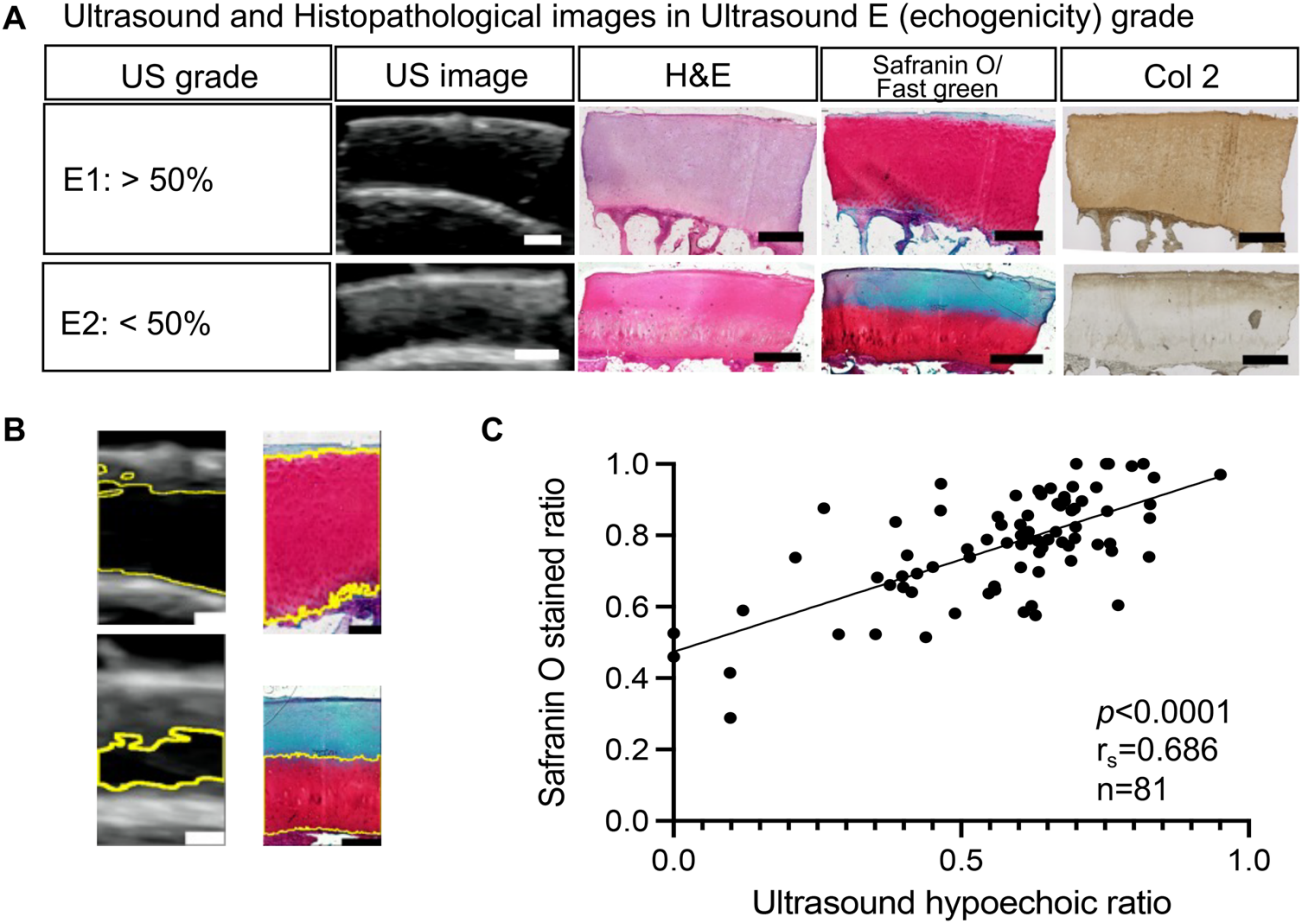
Ultrasound E (cartilage echogenicity) grade and proteoglycan localization in articular cartilage. (**A**) Ultrasound images of typical cases. Scale bars: 1 mm. (**B**) Comparison of ultrasound hypoechoic area and safranin O-stained regions of the central third of images. Scale bars: 1 mm. (**C**) Spearman’s correlation graph between ultrasound hypoechoic ratio and safranin O-stained ratio in histology. Spearman’s correlation coefficient (r_s_) was 0.686.

Cartilage echogenicity was classified as E1 if the hypoechoic area was > 50% of the total cartilage layer and E2 if it was < 50% (Fig. 2). Kappa statistics (K) between the two examiners were available at 1.0. The H&E staining, safranin O/fast green staining, and type 2 collagen (Col 2) immunostaining showed a difference in extracellular matrix components between the E grades. Compared to E1, the proteoglycan content and Col 2 content decreased in E2 (Fig. 2A).

The correlation coefficient between the percentage of hypoechoic areas on US images and the safranin O-stained areas on safranin O staining images in 81 specimens selected from S1 grade specimens was 0.686 (*p* < 0.0001) (Fig. 2B,C).

### Relation between US grading and OARSI OA cartilage histopathology evaluation system

**Figure 3:**
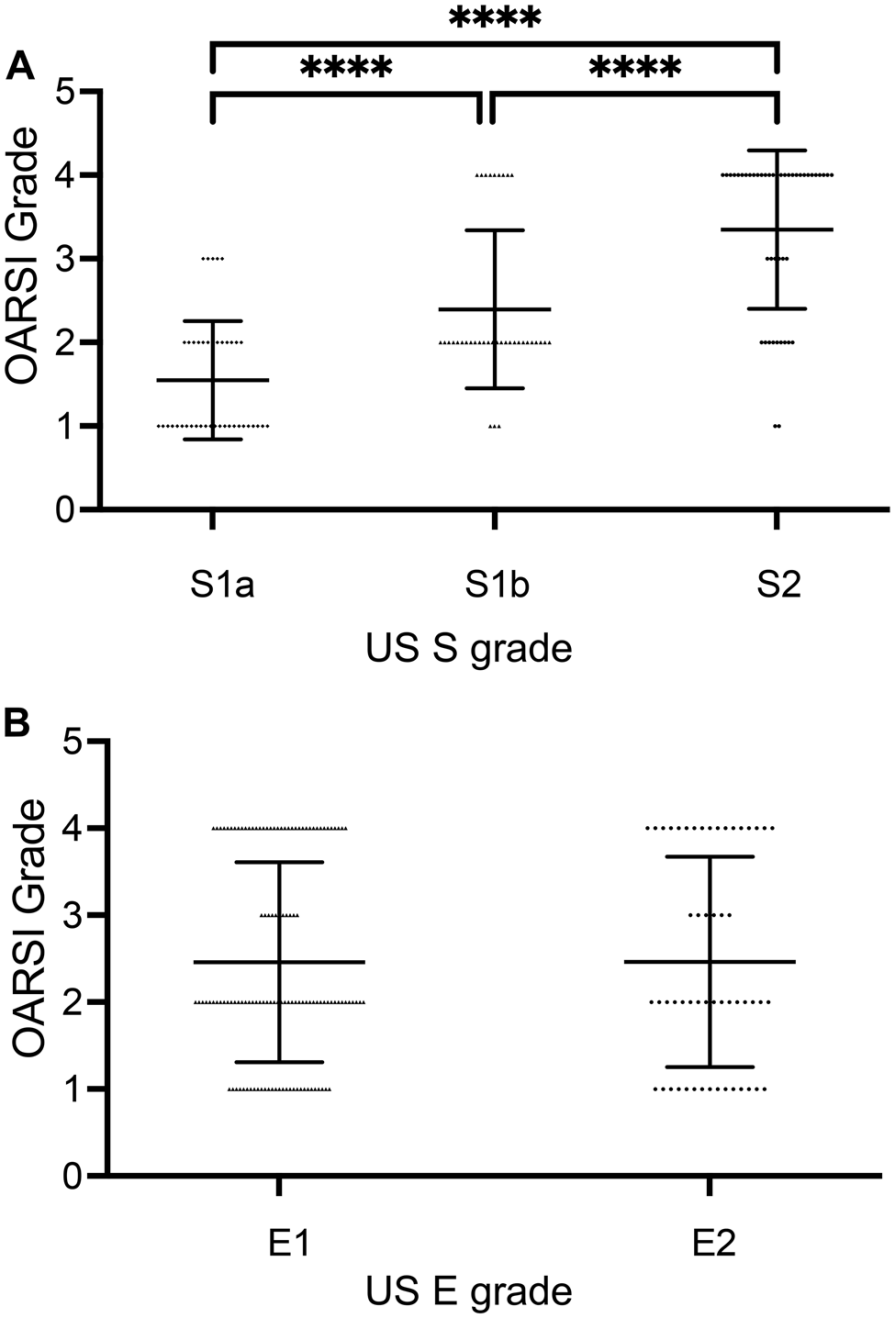
Ultrasound grade and the OARSI osteoarthritis cartilage histopathology assessment system. The relation of OARSI grade with (**A**) US S grade, (**B**) US E grade. The mean OARSI grade in each US grade (mean ± SD). ****p < 0.0001.

The OARSI grades of 126 specimens from 22 donors were measured. The average OARSI grade (mean ± SD) for each US grade was as follows: 1.5 ± 0.7 (S1a), 2.4 ± 1.0 (S1b), 3.4 ± 1.0 (S2), 2.5 ± 1.2 (E1), and 2.5 ± 1.2 (E2). OARSI grades were significantly related to US grades (*p* < 0.0001) (Fig. 3A). S1a was lower than S1b (*p* < 0.0001) and S2 (*p* < 0.0001). S1b was lower than S2 (*p* < 0.0001) too. On the other hand, the OARSI grade was not related to US E grades (*p* = 0.989) (Fig. 3B).

### US grade and OA-related gene expression

**Figure 4:**
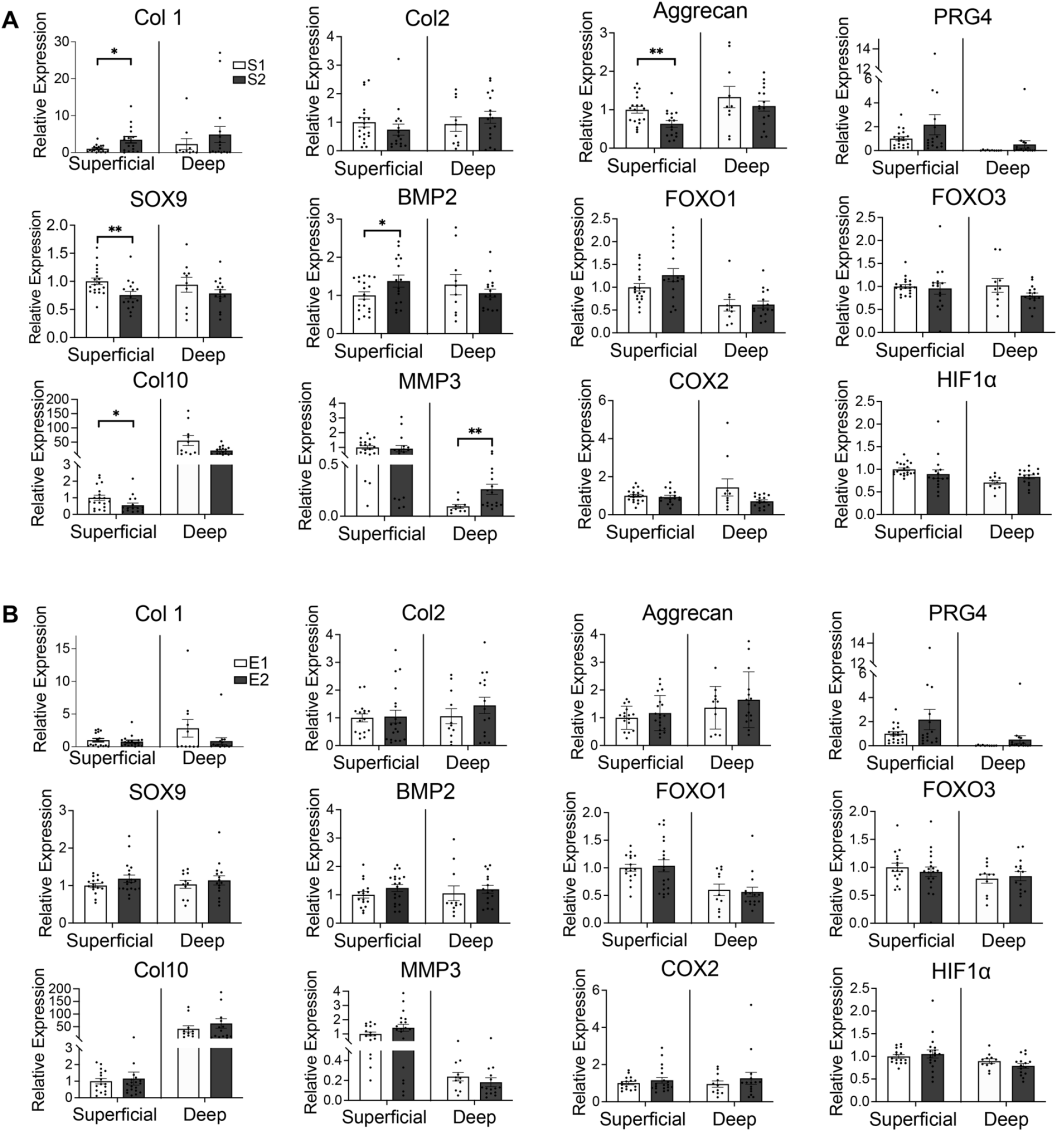
Relationship between ultrasound grade and OA-related gene expression in the articular cartilage. (**A**) Comparison between ultrasound S1 and S2 grades. (**B**) Comparison between ultrasound E1 and E2 grades. Bars are show the mean ± SEM. *p < 0.05, **p < 0.01.

In the superficial layers, Col 10 (1.8-fold, *p* = 0.038), aggrecan (1.6-fold, *p* = 0.006), and SRY-box transcription factor 9 (SOX9) (1.3-fold, *p* = 0.008) were highly expressed in S1 than in S2, whereas Col 1 (0.3-fold, *p* = 0.015) and bone morphogenetic protein 2 (BMP2) (0.7-fold, *p* = 0.043) were less expressed. In the deeper layers, MMP3 (2.0-fold, *p* = 0.005) was more highly expressed in S2 than in S1 (Fig. 4A). Echogenicity grading showed no significant difference in gene expression (Fig. 4B).

### US grade and the response to IL-1ß stimulation

**Figure 5:**
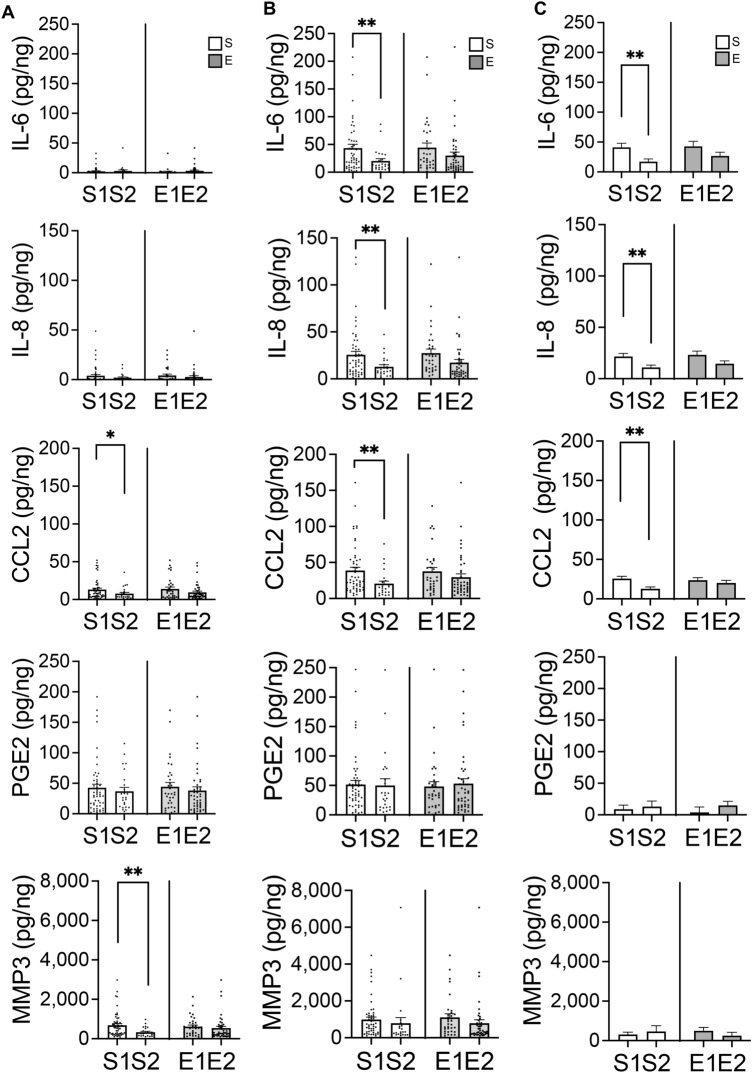
Ultrasound grade and response to IL-1ß stimulation. The sample sizes for each group were: S1(n = 53), S2(n = 24), E1(n = 33), E2(n = 44). (**A**) Protein concentration in the supernatant before addition of IL-1ß. (**B**) Protein concentration in the supernatant after addition of IL-1ß. (**C**) Change in protein concentration in supernatant. Bars are show the mean ± SEM. *p < 0.05, **p < 0.01, ***p < 0.001.

Without IL-1ß stimulation, the concentrations of CCL2 (*p* = 0.039) and MMP3 (*p* = 0.001) proteins in the supernatant were significantly higher in S1 than in S2 (Fig. 5A). After IL1ß stimulation, the concentration of IL-6(*p* = 0.005), IL-8(*p* = 0.004), CCL2(*p* = 0.003) were significantly higher in S1 than S2, whereas the concentration of PGE2 and MMP3 had no difference between US grade (Fig. 5B). The concentrations of PGE2 (*p* = 0.001), MMP3 (*p* = 0.016), IL-6 (*p* < 0.0001), IL-8 (*p* < 0.0001), and CCL2 (*p* < 0.0001) increased after IL-1ß (5 ng/ml) stimulation (Fig. S1). Comparing the response to IL-1β stimulation, the concentration differences of IL-6 (2.4-fold, *p* = 0.006), IL-8 (2.0-fold, *p* = 0.005), and CCL2 (2.0-fold, *p* = 0.001) in the supernatant of S1 were significantly higher than those in S2 (Fig. 5C).

## Discussion

Several non-pharmacological interventions have been demonstrated to delay the progression of early OA. Therefore, there is a great demand for a simple and sensitive method to screen for early cartilage deterioration. The most important finding of this study is that the US cartilage grading system focusing on the cartilage surface using a standard US reflects the biological characteristics of early OA cartilage.

Early changes in OA include the disruption of superficial collagen and loss of proteoglycans^23^. Therefore, a US grading system based on the surface integrity and internal echogenicity of the cartilage was established. Surface roughness analysis confirmed that US grade could accurately reflect the initial changes in the cartilage surface roughness. Additionally, analysis of safranin O/fast green staining revealed that the changes in proteoglycan content of degenerated articular cartilage were well reflected by cartilage echogenicity on US images. In previous studies, US-assisted techniques such as quantitative US techniques, US biomicroscopy, and US backscatter have shown the potential to detect cartilage surface roughness^24,25^, cartilage thickness^26^, changes in proteoglycan content^27^, and could reflect pathological grade^25,28^. To the best of our knowledge, there have been no reports discussing the relationship between quantitative evaluation of articular cartilage surface roughness and standard ultrasonography. The clinical significance of this study is that it shows that information reflecting the proteoglycan content of articular cartilage can be easily obtained by standard ultrasonography, which is available in general clinical facilities.

Another finding of this study was that the US grade focusing on cartilage surface can effectively reflect the OARSI OA cartilage histopathology assessment, and the gene expression pattern was like that in previous studies. It has been shown that as the pathological grade increases, the genes related to matrix metabolism are changed, with increased expression of Col 1^29^ and MMP3^30^, and decreased expression of aggrecan^29^. In this study, cartilage with an irregular surface in US evaluation also showed higher expression in Col 1 and MMP3, and a lower expression in aggrecan compared with cartilage with a smooth surface. Previous study has shown that the proliferative capacity of chondrocytes decreases with the progression of OA^31^, which was evidenced by the decreased expression of SOX9 in the superficial layer of cartilage with irregular surface in this study. In a previous study, BMP2 was found to be upregulated with OA progression and was mainly expressed in the superficial and middle layers of moderately damaged cartilage^32,33^; this study also showed a higher expression of BMP2 in the superficial layer of moderately damaged cartilage. Although many other gene expressions have been demonstrated to be altered in OA cartilage, there were several genes that did not show differences between US grades in this study, such as Col 2^29^. Since only early and mid OA specimens were included in this study, the results may be different from those of previous reports comparing healthy subjects and severely damaged OA cartilage.

IL-1β, a pro-inflammatory cytokine, has been shown to promote OA progression by increasing inflammatory molecules^34^. In this study, the addition of IL-1ß increased the production of PGE2, MMP3, IL-6, IL-8, and CCL2 from articular cartilage, and for IL-6, IL-8, and CCL2, articular cartilage with a smooth surface was more susceptible to IL-1ß than articular cartilage with a worn surface. Previous studies have shown that cartilage from different anatomical sites respond differently to IL-1ß^35^, and that the superficial layers of cartilage are more vulnerable to IL-1ß than the deeper layers^36,37^. This may be one of the reasons why articular cartilage that is closer to normal has a more pronounced response to IL-1ß stimulation compared to articular cartilage that has degenerative changes on the surface.

The limitations of this study include: First, this study focused on early OA and did not include the evaluation of articular cartilage in the advanced stages of degeneration. Joints with OA often contain articular cartilage at various stages of degeneration, and evaluation of articular cartilage in advanced stages of degeneration is necessary in the future. Second, the samples in this study were obtained from total knee arthroplasty, which may be different from the articular cartilage of early OA patients; it is not practical to study the biological properties of articular cartilage from early OA patients, so evaluation by experiments using large animals may be necessary. Thirdly, as mentioned in the text, the area of the knee joint cartilage that can be evaluated by ultrasonography is limited to the femoral side. Although there are a small number of patients who initially develop degeneration of articular cartilage in the tibia or patella, the femur, especially the medial femoral condyle (MFC), is often the first site of degeneration. Therefore, we believe that the significance of this study from the viewpoint of OA diagnosis is confirmed. Lastly, US examination requires the examiner to have a relatively high level of operating skills^13^. In this study, the agreement between the two trained examiners was excellent according to Kappa statistics. In the future, it is also necessary to train and evaluate the operators before the clinical application of this US grading system.

In the future, it is desirable to first validate the US findings of articular cartilage in a large animal model. The use of an animal model of articular cartilage degeneration would reduce the limitations imposed by specimen selection and could bring additional new information to the results of this study. Furthermore, a clinical study focusing on preoperative US evaluation of patients would be very important. Investigating the relationship between preoperative US findings and specimens at the time of surgery would be useful in applying the results of this study to clinical medicine.

In conclusion, US assessment focusing on articular cartilage surface integrity reflected the biological characteristics of articular cartilage in the early stages of degeneration, including the OARSI histopathology grading system, gene expression, and response to IL-1ß (a pro-inflammatory cytokine). The ability to sensitively delineate the early stages of articular cartilage degeneration may be suitable for screening OA, and for frequent longitudinal assessment of the natural course and after therapeutic intervention. The results of this study suggest that standard ultrasonography can be used as a powerful tool in the field of OA treatment and basic articular cartilage research.

## Materials and methods

### Ethics statement

This study was conducted using human osteochondral tissues, which was approved by the Osaka University Institutional Ethical Committee (approval ID: 16,085-4). Written informed consent was obtained from all the subjects, and all the methods were performed in accordance with the relevant guidelines and regulations.

### Sample preparation

**Figure 6:**
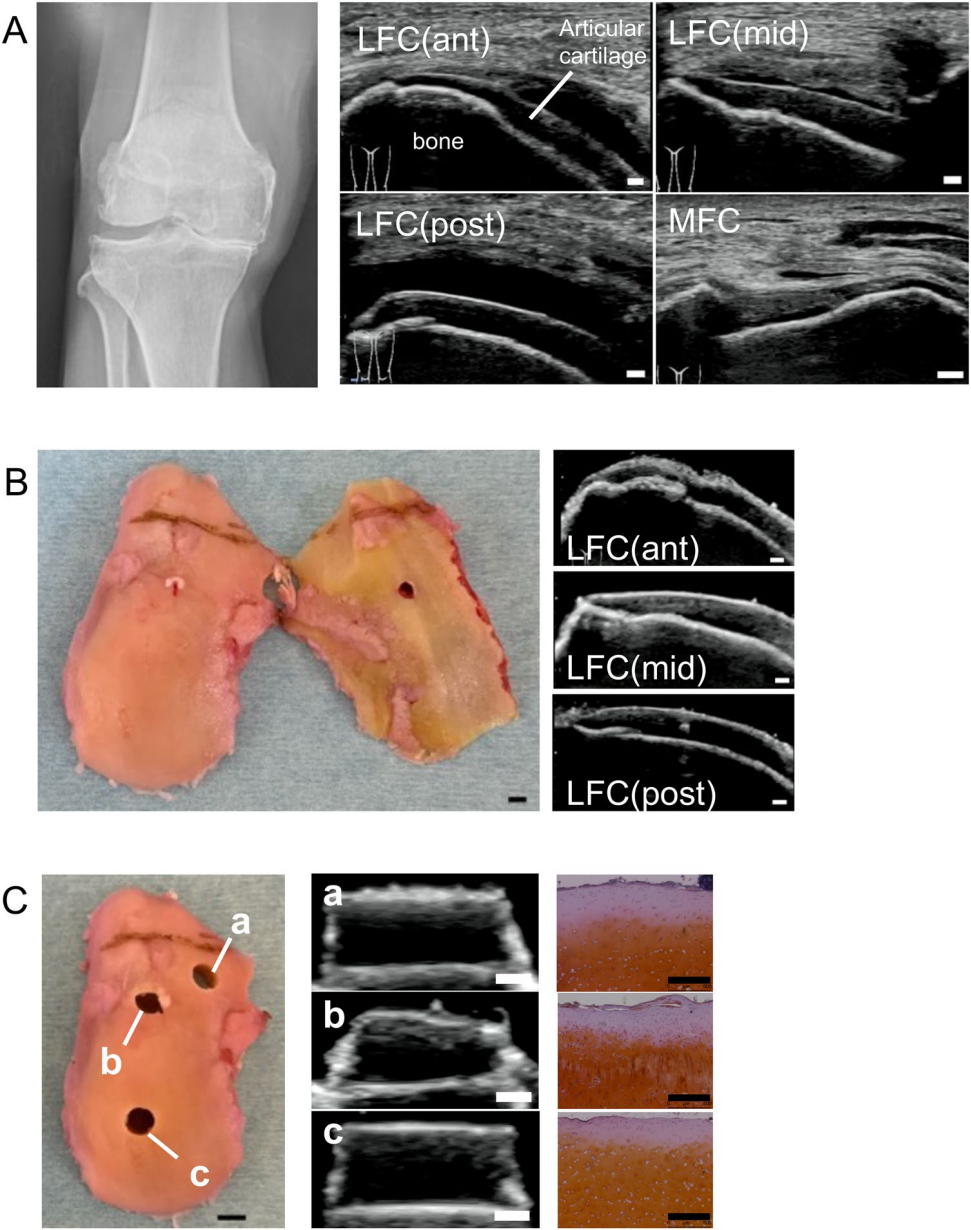
Ultrasound of knee joint articular cartilage reflects various degenerative lesions. (**A**) Plain radiograph and ultrasound images before surgery. The ultrasound images depicted the distal medial femoral condyle (MFC) and three different positions of the lateral femoral condyle (LFC). ant; anterior, mid; middle, post; posterior. (**B**) Ultrasound images of three different positions of the surgically resected specimen. (**C**) Osteochondral columns harvested from three different locations. Scale bars: 1 mm (US), 500 μm (SO), 5 mm (photo).

Human osteochondral specimens were obtained from the lateral femoral condyle (LFC) of the patients diagnosed with OA (N = 22; 19 females, 3 males) within 48 h after total knee arthroplasty. The age of the donors ranged from 66 to 85 years old (mean ± standard deviation: 76.6 ± 5.3 years old). All specimens were obtained from the patients with a certain amount of articular cartilage remaining in the LFC (Fig. 6). Specimens (n = 203) including whole cartilage layers and subchondral bone were drilled using a disposable biopsy punch (diameter = 5/6 mm; Kai Medical, Solingen, Deutschland) under sterile conditions. During drilling, the tissues were rinsed with phosphate-buffered saline (FUJIFILM Wako Pure Chemical Corporation, Chuo-ku, Osaka, Japan) containing 1% penicillin–streptomycin. After the US measurements, the 6 mm diameter specimens (n = 126) were immediately frozen at − 80 °C and used for gene expression and histological analyses. From the 5 mm diameter specimens (n = 77), the subchondral bones were removed, and the cultures were started in Dulbecco’s Modified Eagle’s Medium (DMEM)—high glucose (D6429) (Sigma-Aldrich) containing 1% penicillin/streptomycin at 37 °C and 5% carbon dioxide.

### US measurement and grading

An US evaluation was performed by two examiners using a SONIMAGE HS1 (Konica Minolta Japan, Inc., Tokyo, Japan) equipped with a high-frequency linear probe (HL18-4), ensuring that the US beam was perpendicular to the surface of the cartilage and the boundary between the cartilage and the subchondral bone. The agreement between the two examiners (have 13 years and 3 years of experience in musculoskeletal US respectively) for US evaluation was examined using the Kappa statistics (K), which was interpreted as poor (0.00–0.20), fair (0.21–0.40), moderate (0.41–0.60), good (0.61–0.80), and excellent (0.81–1.00). The percentage of hypoechoic areas throughout the cartilage layer was measured using ImageJ with respect to the central third of the US image of each specimen. During ultrasonography, the specimens were immersed in PBS containing 1% penicillin/streptomycin at room temperature.

### Histological analysis

Frozen Sects. (10 μm) of osteochondral columns were prepared using the Kawamoto’s film method^38^ and subjected to hematoxylin and eosin (H&E) staining, safranin O/fast green staining, and immunostaining. For the central third of the safranin O/fast green stained images, the percentage of the area stained with safranin O to the total cartilage layer was calculated using ImageJ. Histopathological evaluation of OA research society international (OARSI) osteoarthritic cartilage was performed as previously reported^39^. For immunostaining, frozen sections were incubated in permeability buffer (1X PBS/0.2% Triton X-100) for 10 min, 0.3% H_2_O_2_ in 1 × PBS for 30 min, and Blocking One Histo (06,349–64; Nacalai Tesque, Inc., Kyoto, Japan) for 30 min at room temperature. Sections were then incubated overnight at 4 °C with the primary antibodies, such as goat anti-type I collagen (1:200; 1310–01; SouthernBiotech, Birmingham, AL, USA) and goat anti-type II collagen (1:200; 1320–01; SouthernBiotech, Birmingham, AL, USA). Immune complexes were detected using anti-goat IgG H&L (HRP) (1:400; ab97110; Abcam, Boston, MA, USA) and ImmPACT DAB (SK-4105; Vector Laboratories, Burlingame, CA, USA). Images were obtained using a DMi8 (LEICA) microscope or an Olympus BX53 microscope.

### Surface roughness measurement

The thawed specimens were subjected to surface roughness (Ra) analysis using a confocal laser scanning microscope (VK-X200; Keyence Corporation, Osaka, Japan). The measurement was based on the JIS B0601:2001 (ISO 4287:1997) surface texture parameter. Ra (arithmetical mean height; μm) indicates the average of the absolute value of the height of each point along the reference length, which is defined as $\mathrm{Ra}=\frac{1}{\mathrm{N}}{\int }_{0}^{\mathrm{N}}|\mathrm{Z}(\mathrm{x})|\mathrm{dx},$ where N is the defined length, Z is the absolute value of the height of the points, and x is the measurement unit of the X stage. For each specimen, a surface section of 1420 × 1065 μm was scanned. Within each surface section, six linear tracks (1063 μm each) were measured, and the profile filter had cutoff wavelengths λs = 25 μm and λc = 8 μm. The mean Ra value of the tracks was used to represent the surface roughness of the specimens.

### RNA extraction and quantitative real-time polymerase chain reaction (PCR)

Articular cartilage is highly organized structure and can be divided into 3 distinct zones, the superficial, middle and deep zones. Referring to the previous research^40^, we divided cartilage specimens into upper and lower parts according to cartilage thickness, which are named superficial layer and deep layer. According to the histopathology of cartilage^39^, the superficial layer is considered as the superficial zone and part of the middle zone, and the deep layer is considered as part of the middle zone and the deep zone. Firstly, the height of each cartilage specimen was carefully measured and the number of 20 μm thickness cryosections that can be cut into was calculated. Then, cartilage specimens were cut into 20 μm thickness cryosections in a cryostat (CM1860 UV; Leica Biosystems, Wetzlar, Germany) and separated into equal amounts of superficial and deep layers based on the calculated number. Total RNA of each specimen was extracted using Trizol (Invitrogen; Thermo Fisher scientific, Waltham, MA, USA) and a PureLink™ RNA Mini kit (Ambion; Thermo Fisher scientific, Waltham, MA, USA), and reverse transcribed into cDNA using a high-capacity RNA-to-cDNA Kit (Applied Biosystems; Thermo Fisher scientific, Waltham, MA, USA). Quantitative PCR was performed using Power SYBR Green Master Mix (Thermo Fisher scientific, Waltham, MA, USA) and QuantStudio 7 Pro Real-Time PCR System (Thermo Fisher Scientific, Waltham, MA, USA), according to the manufacturer’s instructions. The nucleotide sequences of the primers are described in Supplementary Table 1. Target gene expression was normalized to that of the reference gene glyceraldehyde-3-phosphate dehydrogenase (GAPDH).

### IL-1β stimulation

IL-1β (5 ng/ml; R&D systems, Minneapolis, MN, USA) was added 24 h after the start of culture. The medium was collected 24 h before and after the addition of IL-1β, then used for protein concentration measurement. Twenty-four hours after stimulation, cartilage specimens were harvested for DNA content measurement.

### Quantitative protein analysis using Homogeneous Time-Resolved Fluorescence (HTRF)

An enzyme immunoassay was performed to measure the concentration of prostaglandin E2 (PGE2), matrix metallopeptidase 3 (MMP3), IL-6, IL-8, and C–C motif chemokine ligand 2 (CCL2) using HTRF human PGE2, CCL2, IL-6, and IL-8 assay kits (CIS Bio International SA, Saclay, France) and LANCE Ultra MMP3 (Human) Detection Kit (PerkinElmer, Waltham, MA, USA). The culture supernatant 24 h before adding IL-1ß and 24 h after adding IL-1ß were evaluated with an enzyme immunoassay, and the difference in the protein concentration was calculated. The concentration (pg/ml) was normalized to the total DNA content (pg/ng total DNA).

### DNA Content measurement

The cartilage specimens were digested with Proteinase K solution (Invitrogen; Thermo Fisher scientific, Waltham, MA, USA) overnight at 55 °C. DNA was extracted using a PureLink Genomic DNA Mini Kit (Invitrogen; Thermo Fisher scientific, Waltham, MA, USA), according to the manufacturer’s instructions. DNA concentration was determined fluorometrically using a Qubit 4.0 Fluorometer (Thermo Fisher Scientific, Waltham, MA, USA).

### Statistical analysis

Statistical analysis was performed with analysis of variance (independent-sample T test, Welch’s F test, and ANOVA) and Spearman’s correlation analysis. Results are presented as mean ± standard deviation (SD) or standard error of the mean (SEM). The data were analyzed with SPSS software (version 26.0; IBM, Armonk, NY, USA), and statistical significance was set at *p* < 0.05.

## Supplementary Information

Supplementary Information.

## Data Availability

The data that support the findings of this study are available from the corresponding author upon reasonable request.
