# Supplementary material for: Articular surface integrity assessed by ultrasound is associated with biological characteristics of articular cartilage in early-stage degeneration
Source: Sci Rep. 2022 Jul 13;12:11970. doi: 10.1038/s41598-022-16248-6 (PMC9279397; doi:10.1038/s41598-022-16248-6)
Supplement: Supplementary file 1 — Supplementary Information. [file 41598_2022_16248_MOESM1_ESM.pdf]

Title

Articular Surface Integrity Assessed by Ultrasound is associated with Biological Characteristics of Articular Cartilage in Early-Stage Degeneration.

Authors

Wen Shi[1] MD, Takashi Kanamoto[1*] MD, PHD, Masaharu Aihara[2] MD, Shiro Oka[2] MD, Sanae Kuroda[3] MD, Tsuyoshi Nakai[3] MD, PHD, Takeo Mazuka[4] MD, Keisuke Takenaka[5], Yuji Sato[5] PHD, Masahiro Tsukamoto[5] PHD, Kosuke Ebina[6] MD, PHD, Ken Nakata[1] MD, PHD

Affiliations

[1]Department of Medicine for Sports and Performing Arts, Osaka University Graduate School of Medicine, 2-2 Yamadaoka, Suita, Osaka 565-0871, Japan

[2]Department of Orthopedic Surgery, Aihara Hospital, 3-4-30 Makiochi, Mino, Osaka 562-0004, Japan

[3]Department of Orthopedic Surgery, Itami City Hospital, 1-100 Koyaike, Itami, Hyogo 664-8540, Japan

[4]Department of Orthopedic Surgery, Hannan Chuo Hospital, 3-3-28 Minami-shinmachi Matsubara, Osaka 580-0023, Japan

[5]Joining and Welding Research Institute, Osaka University, 11-1 Mihogaoka, Ibaraki, Osaka 567-0047, Japan

[6]Department of Musculoskeletal Regenerative Medicine, Osaka University Graduate School of Medicine, 2-2 Yamadaoka, Suita, Osaka 565-0871, Japan

Gene		Sequence
GAPDH	Forward	TCTCTGCTCCTCCTGTTCGAC
	Reverse	GTTGACTCCGACCTTCACCTTC
Col 1	Forward	GAGAGTCAGGAGGGGAGCTT
	Reverse	GCTTTTCCCAGGAGGATTTC
Col 2	Forward	TTCAGCTATGGAGATGATGACAATC
	Reverse	AGAGTCCTAGAGTGACTGAG
Col 10	Forward	ATG CTG CCA CAA ATA CCC TTT
	Reverse	GGT AGT GGG CCT TTT ATG CCT
Aggrecan	Forward	GTGGAATGCAGAGGTYY
	Reverse	ACAGCTGGGGACATTAGTGG
PRG4	Forward	TCCATTCAGTCCATCTCC
	Reverse	TGTCCAGTTAGTCCTCCAAATCCT
FOXO1	Forward	AGGGTTAGTGAGCAGGTTACAC
	Reverse	CTGCACACATTGGGCAAACA
FOXO3	Forward	CATGAGAAGTTCCCCAGCGA
	Reverse	GTGTCAGTTTGAGGGTCTGCT
MMP3	Forward	TCCTACTGTTGCTGTGCGTG
	Reverse	AGGTTCATGCTGGTGTCCTC
COX2	Forward	AGGGTTGCTGGTGGTAGGAA
	Reverse	GGTCAATGGAAGCCTGTGATACT
BMP2	Forward	TCCTGAGCGAGTTCGAGTTG
	Reverse	TCTCCGGGTTGTTTTCCCAC
HIF1α	Forward	CTGACCCTGCACTCAATCAA
	Reverse	TCCATCGGAACTAGGTG
SOX9	Forward	TACGACTACACCGACCACCA
	Reverse	TCAAGGTCGAGTGAGCTGTG

Supplementary Table 1: Primers used for RT-qPCR

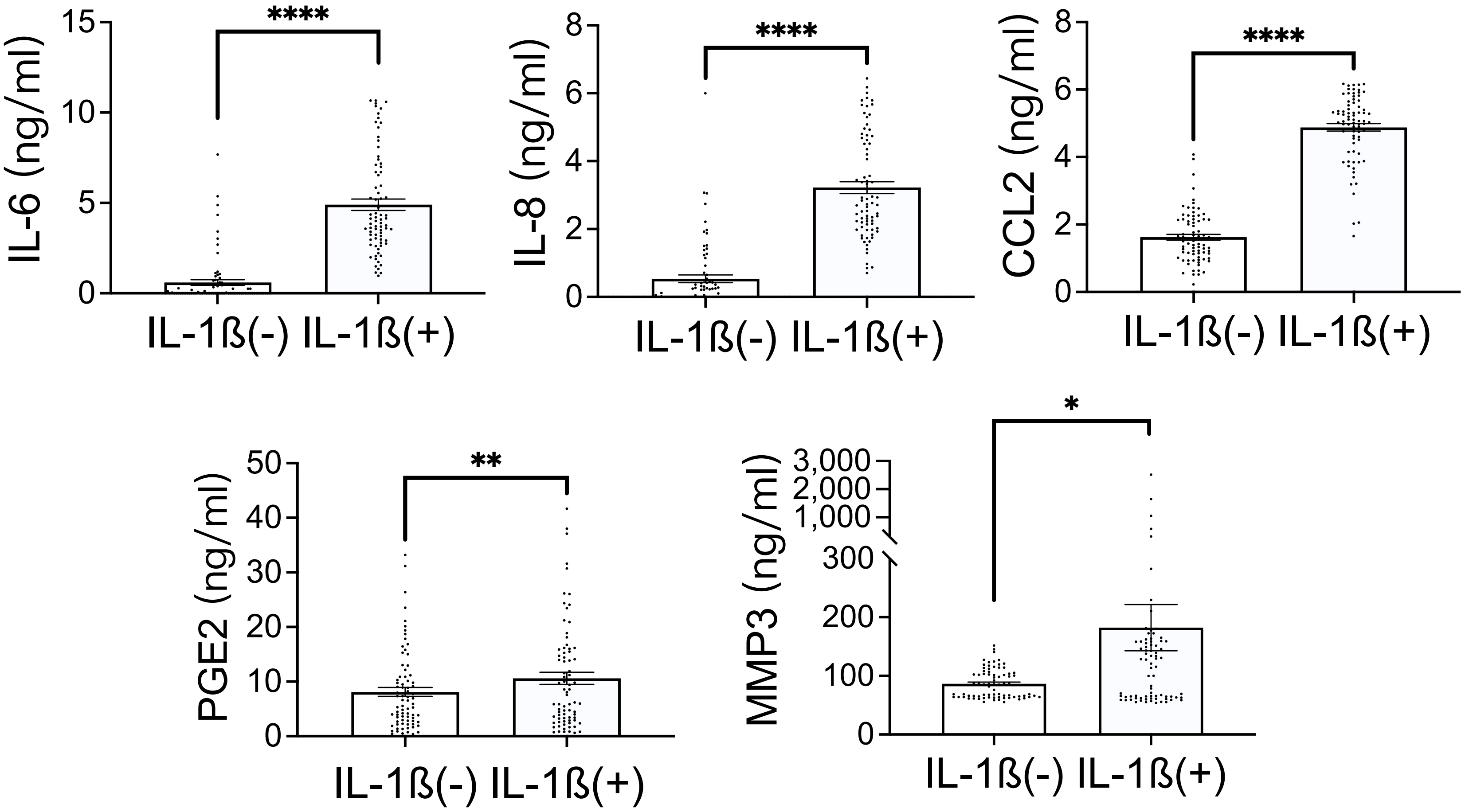

Supplementary Figure 1:

Comparison of protein concentration in the supernatant before and after addition of IL-1ß.

Bars are show the mean ± SEM. *p < 0.05, **p < 0.01, ****p < 0.0001.
